# The role of interleukin-18 and interleukin-18 binding protein in K/BxN serum transfer-induced arthritis

**DOI:** 10.3389/fimmu.2023.1215364

**Published:** 2023-06-21

**Authors:** Sebastien Fauteux-Daniel, Laura M. Merlo Pich, Charlotte Girard-Guyonvarc’h, Assunta Caruso, Emiliana Rodriguez, Cem Gabay

**Affiliations:** ^1^ Division of Rheumatology, Department of Medicine, Geneva University Hospitals, Geneva, Switzerland; ^2^ Department of Pathology and Immunology, University of Geneva, Faculty of Medicine, Geneva, Switzerland; ^3^ Geneva Centre for Inflammation Research, Geneva, Switzerland

**Keywords:** Still’s disease, K/BxN serum transfer-induced arthritis, interleukin-18, interleukin-18 binding protein, rheumatoid arthritis, systemic juvenile idiopathic arthritis

## Abstract

**Background:**

Interleukin-18 is a proinflammatory cytokine, the activity of which is regulated by its natural inhibitor, IL-18 binding protein (IL-18BP). Elevated circulating levels of IL-18 have been observed in patients with systemic juvenile idiopathic arthritis (sJIA) and adult-onset Still’s disease (AOSD), two conditions associated with dysregulated innate immune responses. This study examines the expression and function of IL-18 and IL-18BP in K/BxN serum transfer arthritis (STA), a model that is uniquely dependent on innate immune responses.

**Methods:**

Naïve and serum transfer-induced arthritis (STA) wild-type (WT) mice were used to examine the articular levels of IL-18 and IL-18BP mRNA by RT-qPCR. The cellular sources of IL-18BP in the joints were determined by using *Il18bp*-*tdTomato* reporter knock-in mice. The incidence and severity of arthritis, including mRNA levels of different cytokines, were compared in IL-18BP or IL-18 knock-out (KO) mice and their WT littermates.

**Results:**

IL-18 and IL-18BP mRNA levels were significantly increased in arthritic as compared to normal joints. Synovial neutrophils, macrophages, and endothelial cells represented the cellular sources of IL-18BP in arthritic joints, whereas IL-18BP production was limited to endothelial cells in non-inflamed joints. The incidence and severity of arthritis were similar in IL-18BP KO and IL-18 KO compared to their WT littermates. Transcript levels of different inflammatory cytokines were not different in the two KO mouse lines compared to WT mice.

**Conclusion:**

Although IL-18 and IL-18BP levels were increased in arthritic joints, our results show that the IL-18/IL-18BP balance is not involved in the regulation of STA.

## Background

Interleukin 18 (IL-18) is a pro-inflammatory member of the IL-1 family of cytokines that was initially identified as “Interferon (IFN)-γ Inducing Factor” (IGIF) because of its characteristic property of inducing IFN-γ production (1). As IL-1β, IL-18 is produced as a biologically inert pro-peptide devoid of a signal peptide. It is cleaved and activated by caspase-1 in response to inflammasome activation (2). IL-18 is released via gasdermin-D pores that also assemble upon caspase-1 cleavage.

The biological activity of IL-18 is regulated by its natural inhibitor, IL-18 binding protein (IL-18BP). In fact, IL-18BP forms high-affinity complexes (KD of 26–50 pM) with IL-18, thus preventing its interaction with its cell surface receptors (IL-18R) (3). IL-18BP is constitutively abundant in mouse and human circulation (respectively in the µg/ml and ng/ml range). Its levels increase substantially during inflammatory conditions (4, 5). Indeed, its production is enhanced by IFN-γ, which, therefore, represents a negative feedback loop (6). Disbalance between IL-18 and IL-18BP has been described as pathogenic in autoinflammatory diseases such as adult-onset Still’s disease (AOSD) and its children counterpart, systemic juvenile idiopathic arthritis (sJIA), macrophage activation syndrome (MAS) (7), and NLRC4 gain-of-function inflammasomopathy (8, 9). The topic of IL-18/IL-18BP balance in autoinflammatory diseases has been recently reviewed (10).

Along with strikingly high levels of IL-18, arthritis is another key clinical manifestation of AOSD and sJIA (7). In fact, serum IL-18 correlates with disease activity, thus suggesting that a functional relation may exist between IL-18 and arthritis (11, 12). Moreover, a phase 2 clinical trial showed that the administration of recombinant human IL-18BP (Tadekinig alfa) in AOSD patients led to significant improvement (11). The administration of Tadekinig alfa also resulted in significant improvement in one case of refractory sJIA and MAS (13). Consistently, increased IL-18 in synovial tissue of rheumatoid arthritis (RA) patients was reported in multiple studies (14–17). In mice, studies have demonstrated the role of IL-18 in the pathogenesis of collagen-induced arthritis (CIA). Indeed, arthritis severity was markedly attenuated in IL-18 knock-out (KO) mice (18), as well as by the administration of neutralizing anti-IL-18 antibodies and recombinant human IL-18BP (19). More recently, IL-18 KO mice were reported to have a milder form of K/BxN serum transfer arthritis (STA) (20).

As opposed to CIA, K/BxN STA is uniquely dependent on innate immune responses (21). Our study aimed to explore the expression and function of IL-18 and IL-18BP in K/BxN STA. We used a recently generated knock-in (KI) mouse line, in which the *tdTomato* reporter gene was inserted within the *Il18bp* gene allowing for co-expression (22). We observed that expression of *Il18bp* mRNA was enhanced in arthritic joints and that neutrophils, endothelial cells, and macrophages were the main cellular sources of IL-18BP in the inflamed joints. Using IL-18BP and IL-18 KO mice, we showed that neither IL-18 nor IL-18BP was required to control the incidence and severity of arthritis.

## Methods

### Mice

**Figure 1:**
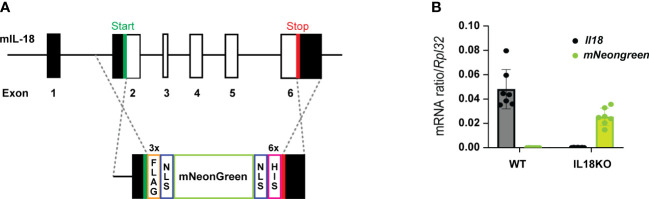
IL-18 KO Neongreen reporter mice express mNeongreen instead of IL-18. **(A)** Schematic representation of insertion of the mNeongreen cassette in the mouse IL-18 gene as performed during the creation of IL-18 KO Neongreen reporter mice. Black rectangles represent noncoding exons. White rectangles are coding exons. NLS = nuclear localization signal. **(B)** Level of expression, relative to *Rpl32*, of *Il18* (in black) and *mNeongreen* (in green) mRNA, in the right ankle of 7 WT and 7 IL-18 KO Neongreen reporter mice, 7 days after the first K/BxN IgG injection.

The generation of IL-18BP KO (C57BL/6N Il18bp^tm1.1(KOMP)Vlcg^) mice has been previously described (Girard-Guyonvarc’h C. 2018). Knock-in (KI) *Il18bp tdTomato* reporter mice were created by Ingenious Targeting Laboratory (Ronkonkoma, NY, USA), as described (22). Briefly, a *tdTomato* reporter gene with two additional nuclear localization sequences was inserted immediately upstream of the coding region of the *Il18bp* gene. The reporter is followed by an A2 self-cleavable peptide, causing independent production of tdTomato and IL-18BP proteins. *Il18bp* gene targeting was performed by homologous recombination in iTL BF1 (C57BL/6 FLP) embryonic stem cells (ES). IL-18 KO Neongreen reporter mice were generated using CRISPR/Cas9 technology on C57BL/6J ES in the VIB Department of Molecular Biomedical Research (Ghent. Belgium). Codon optimized 3x FLAG followed by SV40-NLS and SV40-NLS followed by 6-His tag sequences were respectively N-terminally and C-terminally pasted to the mNeongreen sequence. The megamer was further flanked by two homology sequences for better insertion into the IL-18 gene. After selection of guide RNAs as closest as possible to the IL-18 gene start and stop codons, all IL-18 sequences between start and stop codon were replaced by the mNeongreen cassette (**Figure 1A**). Lack of IL-18 mRNA expression was checked by RT-qPCR in various organs from IL-18 KO mice (**Figure 1B** and **Supplementary Figure 1**). Genotyping on total DNA obtained from the offspring ear biopsy was performed. Heterozygous IL-18BP KO, IL-18 KO, and IL-18BP-tomato+/KI mice were bred in the dedicated area of the Geneva University School of Medicine (Switzerland) animal facility to obtain respectively homozygous IL-18BP KO, IL-18 KO, or IL-18BP-tdTomato KI mice and their co-housed WT littermates. The transgenic mice did not present any phenotypic alteration.

### Purification of the total IgG fraction from K/BxN serum and induction of arthritis

Arthritic K/BxN mice were generated by crossing KRN mice with NOD/Lt mice. The total IgG fraction from the K/BxN serum was purified, as described (23). The flowthrough and elution were then compared to unpurified serum in native or reducing condition electrophoresis (**Supplementary Figure 2**). Recipient mice were injected intraperitoneally with 200 μl of purified KBxN IgG at days 0 and 2, and sacrificed at day 7. Control mice were injected with equal volume of PBS. The development of arthritis was assessed daily, and the severity of arthritis was scored in a blinded fashion for each paw on a four-point scale, in which 0 = normal appearance, 1 = localized edema/erythema over one surface of the paw, 2 = edema/erythema involving more than one surface of the paw, and 3 = marked edema/erythema involving the whole paw. The scores of all four paws were added for a composite score.

### Reverse transcription-quantitative polymerase chain reaction

Right ankles were frozen in liquid nitrogen immediately after sacrifice and stored at −80°C until use. On ice, we added a stainless-steel bead and 700 μl of TRIzol (Invitrogen Life Technologies Carlsbad, CA) to the joints that were then disrupted with TissueLyser 6 min at 30 Hz and centrifuged for 10 min at 14000*g* at 4°C. Supernatants were placed in a new tube with 300 µl of chloroform, vortexed, incubated 3 min at room temperature, and centrifuged for 15 min at 9,660*g* at 4°C. Here, the aqueous phase containing RNA was collected in a new tube. The RNA was then further purified with RNeasy columns (Qiagen, Hombrechtikon, Switzerland) and eluted in 20 μl of H_2_O. Total RNA (1 μg) was treated with RQ1 DNase (Promega, Madison, WI, USA) and reverse transcribed using SuperScript II Reverse transcriptase (Invitrogen Life Technologies, Carlsbad, CA, USA). mRNA levels were assessed by qPCR using appropriate primers (**Supplementary Table 1**) and iQ SYBR Green Supermix in the CFX Connect Real-Time PCR Detection System (Bio-Rad Laboratories Inc., Hercules, CA, USA). The annealing temperature was 60°C. Non-reverse-transcribed RNA samples and water were included as negative controls. RNA expression levels were calculated using the comparative method (2^−ΔCt^) for relative quantification by normalization to *Gapdh or Rpl32* gene expression.

### Immunofluorescence staining

Cytospin was performed on peripheral blood and mounted on Superfrost plus glass slides. Briefly, red blood cells were lysed and 10^5^ cells were mounted on glass slides and then fixed using PFA 4%, pH 7.2, for 15 min at room temperature (RT). The primary antibodies rabbit anti-RFP (Abcam; AB_10971665, 1/200) and rat anti-Ly6G (AB_1186105, 1/100) were then added and incubated overnight at 4°C. The primary antibody was rinsed with IHC buffer and with PBS 1×. Autofluorescence was quenched with 50 μl of TrueVIEW Autofluorescence Quenching Kit (Vector, Burlingame, CA, USA) for 3 min at room temperature. The slides were rinsed with PBS for 5 min and then IHC buffer for 5 min. The secondary antibodies were anti-rabbit AF594 (Invitrogen, AB_141637, 1/1000) and anti-rat AF488 (Thermo Scientific, AB_2535794, 1/500), incubated for 30 min at room temperature. The slides were then rinsed with wash buffer for IHC and PBS 1× and finally stained with DAPI (1/1000) in PBS 1× for 10 min at room temperature. A final rinse with PBS 2× was followed by mounting medium. Slides were imaged with an LSM700 confocal microscope (Carl Zeiss Microscopy, Feldbach, Switzerland).

Left ankle joints of *Il18bp-tdTomato* KI and WT littermates were formalin-fixed and paraffin-embedded. Three-micrometer sections were deparaffinized in xylol and rehydrated through graded concentrations of ethanol. Endogenous peroxidase was blocked and tissue sections were boiled in citrate-based antigen retrieval solution. The slides were incubated overnight at 4°C with either rat anti-Ly6G (BioLegend, AB_1186105, 1/200) or goat anti-mouse/rat CD31/PECAM-1 Antibody (RnD; AB_2161028, 1/100) and with anti-RFP (Abcam; AB_10971665, 1/200) antibodies. Joints from IL-18BP-tdTomato KI and WT littermates were embedded in OCT and were stained for F4/80. The cryo-sections stored at −20°C were thawed and washed with PBS 1× and wash buffer IHC. The slides were stained overnight at 4°C with anti-F480 (BioLegend, AB_893504, 1/200 in DAKO Diluent) and anti-RFP (rabbit, Abcam, AB_10971665, 1/200) antibodies. The slides were then washed with IHC buffer and treated with autofluorescence quenching solution TrueVIEW Autofluorescence Quenching (Vector, Burlingame, CA, USA) for 3 min at room temperature. The slides were washed with PBS 1× and stained with secondary antibodies: anti-Rat IgG Alexa Fluor 488 (Thermo Scientific, AB_2535794 1:500 in Dako Diluent) and anti-rabbit IgG Antibody Alexa Fluor 594 (Invitrogen, AB_141637, dilution 1:1000) for 30 min at room temperature. After washing with PBS 1× and IHC buffer, DAPI (1:1000 in PBS) was added for 10 min at room temperature. The slides were washed with PBS 1× before mounting medium was applied and the images were obtained with confocal LSM700, objective 63×.

### Statistical analysis

Results are represented as individual values, except for variations from baseline body weights, that are expressed as mean percentage ± SEM. Unpaired two-tailed Mann–Whitney test has been used to test statistical significance, or a simple linear regression was used as indicated in the figure legends. *p*-values <0.05 were considered significant. Only statistically significant differences are shown. All plots and statistical analyses were performed using GraphPad Prism 9 software (GraphPad Software, La Jolla, CA). Adobe Illustrator (Adobe, San Jose, CA) was used to reformat graphics and figures.

## Results

### IL-18 and IL-18BP expression in the K/BxN STA joints

**Figure 2:**
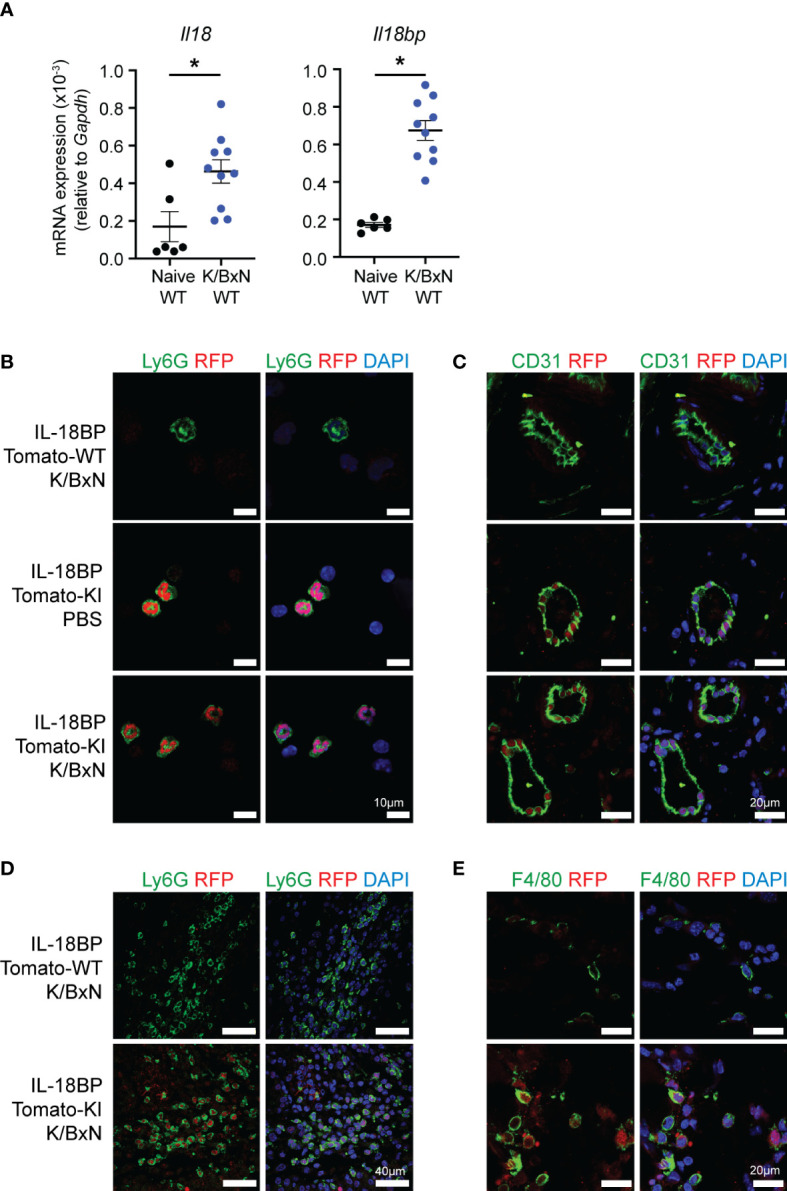
Neutrophils express IL-18BP in K/BxN STA joints. **(A)** Determination of *Il18* and *Il18bp* mRNA levels in the right ankle joints of naïve (black = 6) and K/BxN arthritic (blue = 10) littermates, after 7 days from the first K/BxN IgG injection, relative to *Gapdh*. Data are shown as the mean ± SEM of values. Statistical analysis was performed using a Kruskal–Wallis test. A significant *p*-value is represented *p ≤ 0.05. **(B–E)** Immunofluorescent staining of **(B)** circulating white blood cells and **(C–E)** left ankle joints of WT and IL-18BP-Tomato-KI mice after 7 days from the first PBS or K/BxN IgG injection. The sections are stained for DAPI (blue) and RFP (red) as well as for endothelial cells using anti-CD31 antibody **(C)**, synovial neutrophils using anti-Ly6G antibody **(D)**, and macrophages using anti-F4/80 antibody **(E)** (green). The scale is present on panels **(A)** 10 μm, **(B)** 20 μm, **(C)** 40 μm, and **(D)** 20 μm.

We determined the mRNA levels of *Il18* and *Il18bp* in the joints of naïve and arthritic mice with K/BxN STA. We observed that *Il18* and *Il18bp* mRNA levels were significantly increased in arthritic joints as compared to the joints of naïve mice (**Figure 2A**). To assess the cellular sources of IL-18BP within the inflamed and control joints, K/BxN IgG or PBS was injected to IL-18bp-tdTomato KI reporter mice and WT controls. Immunofluorescence staining was performed with anti-RFP antibody for *tdTomato* gene reporter detection. Cell-specific counterstaining was performed using anti-Ly6G antibody for neutrophils (**Figures 2B, D**), anti-CD31 antibody for endothelial cells (**Figure 2C**), and anti-F4/80 antibody for macrophages (**Figure 2E**). DAPI staining was used as nuclear marker. **Figure 2B** shows that red nuclear fluorescence consistent with tdTomato positivity is present in circulating neutrophils of both naïve and K/BxN STA mice. Similarly, the *tdTomato* gene reporter is also detected in synovial neutrophils of K/BxN STA, indicating that neutrophils are a source of IL-18BP. Of note, there is a positive correlation between *Il18bp* and *Il1r2* mRNA levels in arthritic joints (**Supplementary Figure 3**). This finding is consistent with previous observations showing that neutrophils are the major cellular source of *Il1r2* in K/BxN STA joints (24). Synovial endothelial cells (**Figure 2C**) and macrophages (**Figure 2E**) also stained positive for *tdTomato* reporter. However, in the case of synovial endothelial cells, the nuclear red fluorescence was also present in naïve conditions, indicating that these cells are the source of basal IL-18BP production in the synovium. Of note, we observed a correlation between *Il18* and *Il18bp* mRNA levels in the joints of mice collected 7 days after STA induction (**Supplementary Figure 3**).

### IL-18BP KO and WT mice show comparable K/BxN STA severity

**Figure 3:**
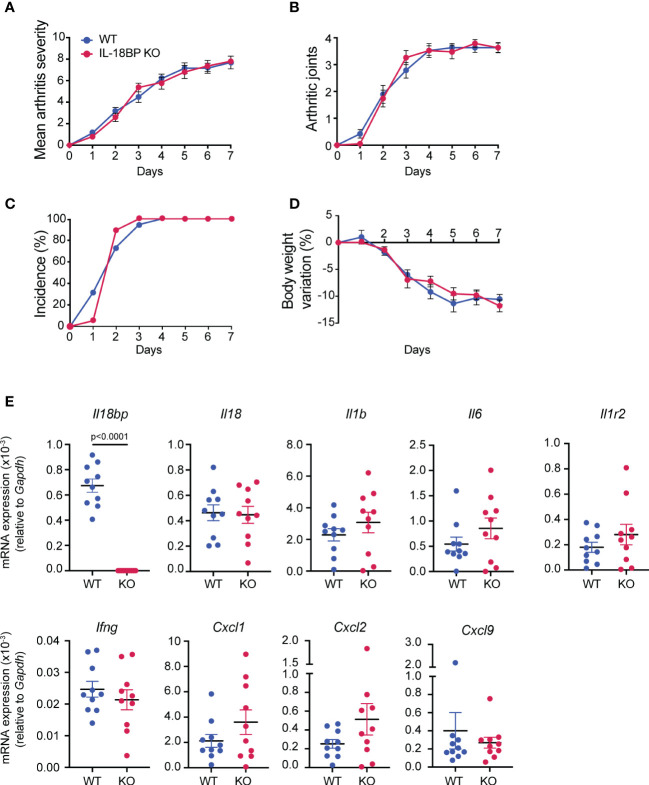
Clinical severity and inflammatory marker expression of K/BxN STA in WT and IL-18BP KO mice. WT (blue, *n* = 20) and IL-18BP KO (red, *n* = 20) littermates were injected i.p. with IgG purified from 150 μl of K/BxN serum on days 0 and 2. Clinical scores were blindly evaluated, daily up to day 7. **(A)** Mean arthritis severity, obtained from the sum of arthritis severity score of all 4 paws on a scale of 0 to 12. **(B)** Number of arthritic paws (arthritis severity score ≥1). **(C)** Incidence of arthritis, seen as percentage of mice with ≥1 joint with arthritis score ≥1. **(D)** Percentage of body weight variation (%). The results are shown as the mean ± SEM. **(E)** Determination of mRNA levels of *Il18bp, Il18, Il1b, Il6, Il1r2, Ifng, Cxcl9, Cxcl1*, and *Cxcl2* in ankle joints. Total RNA was isolated from right ankle joints of WT (blue, *n* = 10) and IL-18BP KO (red, *n* = 10) at day 7 after the first K/BxN IgG injection for qRT-PCR analysis. Results represent *Il18bp, Il18, Il1r2, Il6, Ify, Il1b, Cxcl9, Cxcl1*, and *Cxcl2* mRNA expression levels relative to *Gapdh* mRNA levels. Data are shown as the mean ± SEM of values. Statistical analysis was performed using a Mann–Whitney test. *p*-values not shown signify a lack of statistical significance.

Having established that the local expression of IL-18 and IL-18BP is increased in arthritic joints, we first assessed whether IL-18BP is involved in the control of inflammatory responses. K/BxN STA was induced in IL-18BP KO mice (*n* = 20) and their respective WT littermates (*n* = 20) by two injections of K/BxN-purified IgG on days 0 and 2, followed by daily clinical assessment up to day 7. As depicted in **Figure 3**, there was no difference regarding the severity score of arthritis (**Figure 3A**), the number of arthritic paws (**Figure 3B**), the incidence of arthritis (**Figure 3C**), and body weight variation (**Figure 3D**) between IL-18BP KO and WT mice.

To further assess the severity of arthritis, we measured the mRNA levels of a variety of pro-inflammatory cytokines and chemokines, including *Il18bp, Il18, Il1b, Il6, Il1r2, Ifng, Cxcl9, Cxcl1*, and *Cxcl2* in arthritic joints of IL-18BP KO and WT littermates. **Figure 3E** shows that the mRNA levels of all investigated markers were not significantly different in IL-18BP KO compared to WT mice with the exception of *Il18bp* mRNA levels.

### IL-18 KO and WT mice show comparable K/BxN STA severity

**Figure 4:**
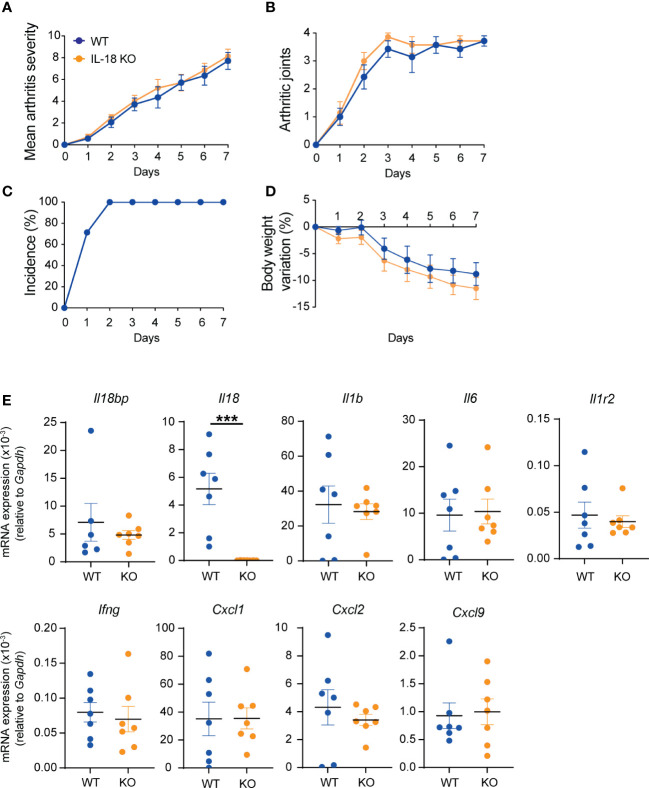
K/BxN STA severity in WT and IL-18 KO littermates. WT (blue, *n* = 7) and IL-18 KO (orange, *n* = 7) littermates were injected i.p. with IgG purified from 150 μl of K/BxN serum on days 0 and 2. Clinical scores were blindly evaluated, daily up to day 7. **(A)** Mean arthritis severity, obtained from the sum of arthritis severity score of all 4 paws on a scale of 0 to 12. **(B)** Number of arthritic paws (arthritis severity score ≥1). **(C)** Incidence of arthritis seen as percentage of mice with ≥1 joint with arthritis score ≥1; WT incidence is identical to IL-18 KO) and **(D)** body weight variation (%). **(E)** Determination of mRNA levels of *Il18bp, Il18, Il1b, Il6, Il1r2, Ifng, Cxcl9, Cxcl1*, and *Cxcl2* in ankle joints. Total RNA was isolated from right ankle joints for qRT-PCR analysis. Results represent mRNA expression levels relative to *Gapdh*. Data are shown as the mean ± SEM of values. Statistical analysis was performed using a Mann–Whitney test. *p*-values not shown signify a lack of statistical significance. *** denotes p ≤ 0.001.

As IL-18BP KO mice did not display a more severe form of K/BxN STA than their WT littermates, we aimed to explore the effects of IL-18 deficiency in this model. K/BxN STA was induced in *n* = 7 IL-18 KO and *n* = 7 WT littermates. As depicted in **Figure 4**, there was no difference in the arthritis severity score, the mean number of arthritic paws, the incidence of arthritis, or the body weight variation between IL-18 KO and WT animals (**Figure 4A**). Similarly, the transcript levels of *Il18bp*, *Il1b*, *Il6*, *Il1r2*, *Ifng*, *Cxcl1*, *Cxcl2*, and *Cxcl9* in the right ankle joint were not different in IL-18 KO and WT mice (**Figure 4B**).

As synovial GM-CSF was previously shown to be produced in response to IL-18 and to contribute to the development of STA (20), we measured the mRNA levels of *Csf2* in the joints of IL-18 KO and WT mice. In accordance with the absence of difference in arthritis severity, *Csf2* mRNA levels were comparable in IL-18 KO mice and WT littermates (**Supplementary Figure 4**).

## Discussion

Cytokines of the IL-1 family play a key role in inflammatory responses. In particular, serum levels of IL-18 are markedly elevated in patients with sJIA and AOSD. Since both sJIA and AOSD are characterized by dysregulated innate immune responses, we decided to investigate the role of the IL-18/IL-18BP balance in an experimental model of arthritis that is uniquely dependent on innate immune responses. Our study showed that *Il18* and *Il18bp* mRNA levels are increased in K/BxN STA joints as compared to non-inflamed joints. Endothelial cells are a source of basal production of IL-18BP in the synovium, while recruited neutrophils, activated macrophages, and endothelial cells participate in the production of IL-18BP during articular inflammation. Despite previous reports of human fibroblast expressing IL-18BP in the context of RA, we could not detect the *Il18bp*-*tdTomato* reporter in synovial fibroblast (25–27). However, the absence of RFP (tdTomato reporter) staining does not rule out the expression of IL-18BP by other synovial cells such as fibroblasts but may merely reflect the limit of sensitivity of our techniques. By using IL-18BP- and IL-18-deficient mice, we observed that the incidence and severity of K/BxN STA were independent of the IL-18/IL-18BP balance. In view of other IL-1 cytokines, these results are in marked contrast with the critical role of IL-1β in the development of K/BxN STA, whereas both IL-33 and IL-36 were devoid of any effect in this model of arthritis (23, 24, 28).

The role of IL-18 was examined in two models of arthritis depending on innate immune responses. In *Streptococcal* cell wall-induced arthritis, the use of neutralizing rabbit anti-IL-18 antibodies significantly attenuated the severity of joint swelling and the local production of pro-inflammatory cytokines and prevented the inhibition of cartilage proteoglycan synthesis. Of note, this effect was independent of IFN-γ (29). Using the K/BxN STA model, IL-18 was recently shown to contribute to the severity of arthritis and intra-articular neutrophil recruitment via the stimulation of synovial NK cells to produce GM-CSF. Indeed, IL-18-deficient mice exhibited attenuated joint inflammation and decreased articular neutrophil recruitment, synovial fluid GM-CSF levels, and GM-CSF-positive synovial NK cells. Of note, IFN-γ did not contribute to the development of K/BxN STA (20). In contrast to these results, we did not observe any significant differences regarding the severity of arthritis, including articular *Csf2* mRNA levels, in IL-18-deficient as compared to WT mice. This discrepancy can be related to differences in environmental conditions of animal facilities and in the genetic background. Regarding the latter, IL-18 KO mice used in the study of Louis et al. were created in a mixed genetic 129xC57BL/6 background (30) and then backcrossed into the C57BL/6J. In the present study, all genetically modified mouse lines, including IL-18-deficient mice, were generated directly in the C57BL/6 background and WT littermates were used in all the experiments.

The role of IL-18 signaling has been examined in models of arthritis dependent on adaptive immune responses such as collagen-induced arthritis and antigen-induced arthritis. The results of most of the studies showed that IL-18 signaling contributes to the development and severity of CIA. Indeed, the administration of either a neutralizing anti-IL-18 antibody or rhIL-18BP significantly attenuated the severity of arthritis and cartilage degradation as compared to placebo-treated mice (19). As compared to WT mice, IL-18-deficient mice showed a reduced incidence and severity of arthritis associated with decreased spleen and lymph node cell proliferation and pro-inflammatory cytokine production in response to *ex vivo* stimulation with bovine type 2 collagen (18). Similarly, IL-18R alpha-deficient mice exhibited an attenuated form of arthritis with reduced synovial CD4 T cell and macrophage infiltration as well as lower serum levels of IL-6, IL-18, TNF-α and IFN-γ as compared to WT mice (31). Conversely, administration of rIL-18 alone or in combination with rIL-12 increased the severity of CIA (32). Administration of mouse IL-18BP as a fusion protein with the Fc portion of murine IgG1 significantly attenuated the clinical and histological scores of arthritis as compared to PBS-treated mice. The proliferation of *ex vivo* antigen stimulated spleen and lymph node cells and circulating anti-collagen IgG1 and IgG2a levels were decreased in IL-18BP-treated mice (33). Overexpression of IL-18BPc by intra-articular adenoviral delivery also attenuated the incidence and severity of CIA in injected knee joints as well as provided additional protection in distal joints (34). As opposed to all the results described above, intravenous administration of an adenovirus encoding soluble IL-18Rβ, which showed IL-18 inhibitory activities, led to the development of exacerbated joint inflammation, bone, and cartilage destruction, despite decreased IFN-γ and IL-4 (35). Of note, IL-18Rβ-treated mice showed enhanced IL-17 production by spleen T cells and reduced circulating regulatory T cells. Moreover, IL-18 was found to be redundant in antigen-induced arthritis in response to an intra-articular injection of bovine serum albumin in mice immunized against this antigen. Indeed, in this model, IL-18 KO mice developed similar arthritis scores and antigen-stimulated T-cell proliferation and IFN-γ production to WT mice (36).

The production and function of IL-18 was also examined in patients with rheumatoid arthritis. IL-18 mRNA and protein are expressed in the rheumatoid synovium with significantly higher levels than in osteoarthritis tissues (14). Serum and synovial fluid levels of IL-18 were higher in RA than in osteoarthritis patients and correlated with disease activity as assessed by the Disease Activity Score 28 (DAS28) (37). In contrast, a cross-sectional study showed that IL-18 levels did not correlate with parameters of disease activity and joint damage as well as disease improvement after methotrexate treatment (38). Despite numerous reports showing the presence of elevated IL-18 in rheumatoid arthritis, it is important to mention that measured IL-18 levels do not necessarily reflect its biological activity. Indeed, commercially available assays do not distinguish free unbound IL-18 from IL-18 complexed with IL-18BP. By using an immunoassay that measures specifically free biologically active IL-18, we were able to show that serum IL-18 levels were not higher in rheumatoid arthritis and psoriatic arthritis patients compared to healthy controls. In marked contrast, free IL-18 levels were significantly increased in patients with AOSD (12) and sJIA with and without MAS (7). Matched serum and synovial fluid levels were examined in 50 patients with JIA, including 24 with oligoarticular JIA, 13 with polyarticular JIA, and 13 with sJIA. IL-18 levels were markedly higher in patients with sJIA than in other subtypes of JIA but did not differ between serum and synovial fluid. IL-18 levels correlated positively with both serum and synovial levels of IL-6 (39). Serum IL-18 was significantly higher in AOSD than RA patients and healthy controls. IL-18 and IL-6 levels correlated with disease activity in AOSD patients. Synovial tissue IL-18 mRNA levels were significantly higher in AOSD patients than in osteoarthritis patients, whereas TNF-α and IL-8 mRNA levels were higher in RA patients than in AOSD patients (40). Whether free IL-18 observed in sJIA and AOSD patients contributes to distal articular manifestation remains to be clarified.

In our mouse model, the *Il18* coding regions were replaced by the *mNeonGreen* reporter gene, which was expressed in different organs at the transcript level in a comparable manner to endogenous IL-18. Unfortunately, we were not able to detect the mNeongreen protein on tissue sections, even in tissues with the highest mRNA expression levels such as in the digestive tract (data not shown). We suppose that the level of gene expression is not sufficiently strong to detect the mNeongreen fluorescence on tissue sections.

## Conclusions

This study showed that despite IL-18 and IL-18BP being induced in the joints during STA, the IL-18/IL-18BP balance is not involved in the pathogenesis of K/BxN STA.

## Data availability statement

The original contributions presented in the study are included in the article/**Supplementary Material**. Further inquiries can be directed to the corresponding author.

## Ethics statement

This research was approved by the Geneva cantonal state authority for animal experimentation under licenses GE121/33936 and GE229/35080.

## Author contributions

CG raised funding. SF-D, LMMP, CG-G, and CG wrote the manuscript. All authors reviewed the manuscript. SF-D and CG designed the study. Acquisition of data was performed by SF-D, LMMP, CG-G, AC, and ER. Data analysis and interpretation by SF-D, LMMP, CG-G, ER, and CG. SF-D and ER performed longitudinal follow-ups. ER and LMMP performed IF and confocal analysis. SF-D, CG-G, AC, and ER characterized the IL-18 reporter mice. SF-D, LMMP, CG-G, AC, and ER performed RT-qPCRs. All authors contributed to the article and approved the submitted version.
