# Supplementary material for: The role of interleukin-18 and interleukin-18 binding protein in K/BxN serum transfer-induced arthritis
Source: Front Immunol. 2023 Jun 21;14:1215364. doi: 10.3389/fimmu.2023.1215364 (PMC10320286; doi:10.3389/fimmu.2023.1215364)
Supplement: Supplementary file 1 [file DataSheet_1.pdf]

Target	GenBank	Primer	Primer sequence 5’->3’
Il18	NM_008360.1	Il18 fwd	CAGGCCTGACATCTTCTG
		Il18 rev	CTGACATGGCAGCCATT
Il18bp	NM_010531.1, XM_017321987.1	Il18bp fwd	ACATCTGCACCTCAGACAACT
		Il18bp rev	TGGGAGGTGCTCAATGAAGGAACCA
Il1r2	NM_010555	Il1r2 fwd	GGTGCGGACAATGTTCATCTTG
		Il1r2 rev	GGGAACTGCTGGAGATGTCGGAGTG
Il6	NM_031168.1	Il6 fwd	TGAACAACGATGATGCACTTGCAGA
		Il6 rev	TCTGTATCTCTCTGAAGGACTCTGGCT
Il1b	NM_008361.3	IL1b fwd	TGTGAAATGCCACCTTTTGA
		Il1b rev	GTGCTCATGTCCTCATCCTG
Ifng	NM_008337.4	Ifng fwd	CTTCTTCAGCAACAGCAAGGCG
		Ifng rev	ATGCTTGGCGCTGGACCTGTG
Cxcl1	NM_008176.3	Cxcl1 fwd	ACTCAAGAATGGTCGCGAGG
		Cxcl1 rev	GTGCCATCAGAGCAGTCTGT
Cxcl2	NM_009140.2	Cxcl2 fwd	AGGGCGGTCAAAAAGTTTGC
		Cxcl2 rev	CGAGGCACATCAGGTACGAT
Cxcl9	NM_008599.4	Cxcl9 fwd	CGAGGCACGATCCACTACAA
		Cxcl9 rev	AGGCAGGTTTGATCTCCGTT
Csf2	NM_009969.4	Csf2 fwd	CTCACCCATCACTGTCACCC
		Csf2 rev	TGAAATTGCCCCGTAGACCC
Gapdh	NM_001289726.1	Gapdh fwd	AGGCCGAGAATGGGAAGCTTGT
		Gapdh rev	TACTCAGCACCGGCCTCACCC
L32	NM_172086.2	L32 fwd	CACCAGTCAGACCGATATGTGAAAA
		L32 rev	TGTTGTCAATGCCTCTGGGTTT

Table 1: RTqPCR primer sequences.

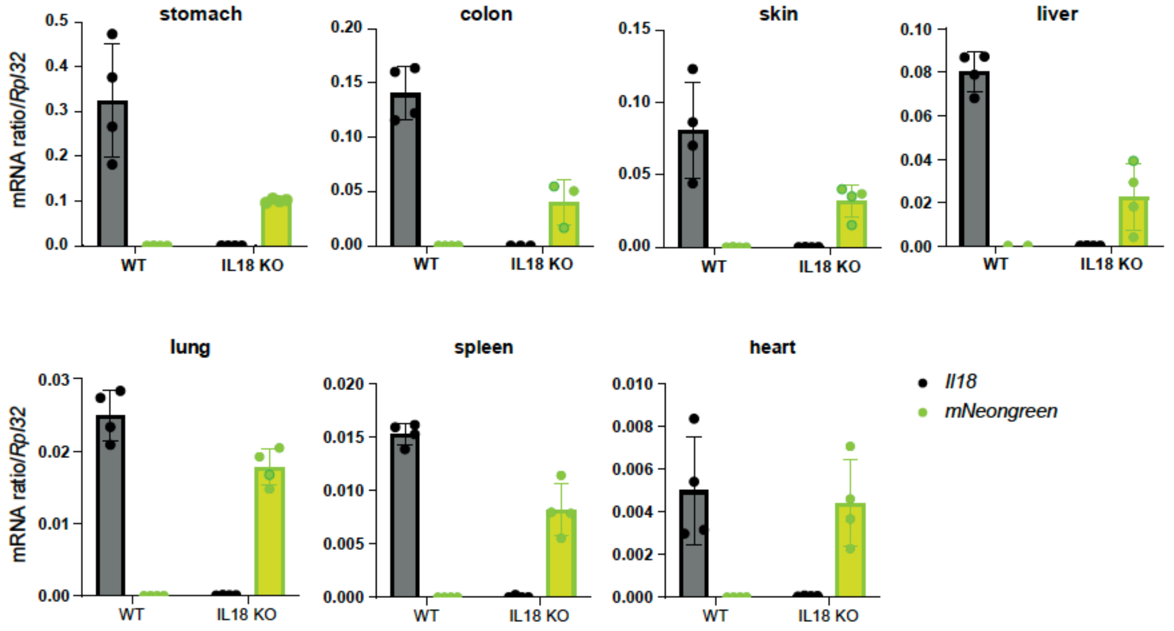

Figure 1. Expression of *Il18* and *mNeongreen* mRNA in organs of IL-18 KO Neongreen reporter vs WT mice. WT (black, n = 4) and IL-18 KO (light orange, n = 4) littermates were injected i.p. with IgG purified from 150µl K/BxN serum on days 0 and 2 then sacrificed on day 7. Total RNA was extracted from stomach, colon, skin, liver, lung, spleen and heart for RT-qPCR analysis. Results represent mRNA expression levels relative to *l32*. Data are shown as the mean ± SEM of values.

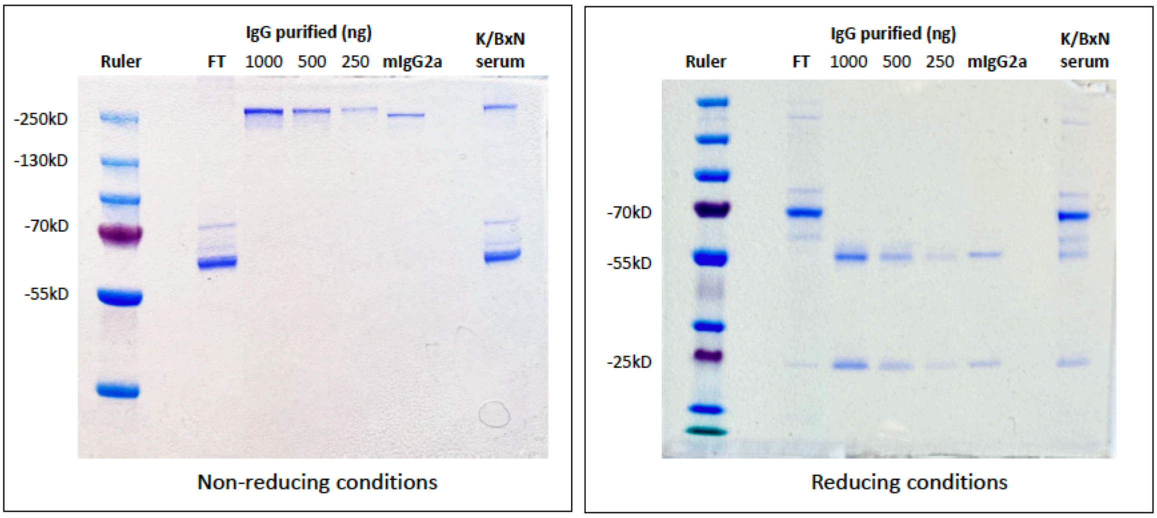

Figure 2: K/BxN IgG purification in both non-reducing and reducing conditions. The purified IgG were run at different concentrations, compared to the flowthrough (FT), a known IgG (mIgG2a) and the original K/BxN serum. Gel composition was 4-12% Bis-Tris and they were run at 100V for 20min then 130V.

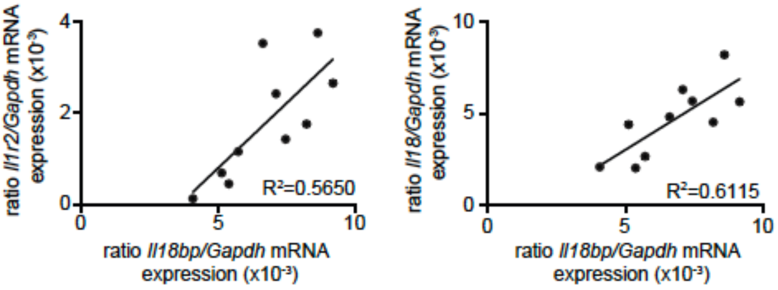

Figure 3: Correlation of mRNA levels in ankle joint. Expression levels of *Il1r2* or *Il18* mRNA were correlated to that of *Il18bp* in WT (n=10) mice after seven days from the first K/BxN purified serum injection.R^2 represents Pearson's coefficient of determination.

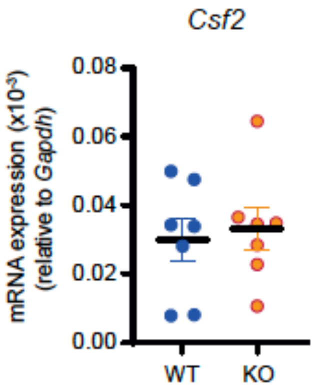

Figure 4: Expression levels of Csf2 mRNA in joint of K/BxN STA IL-18 KO vs WT littermates. Total RNA was isolated from right ankle joints of WT (blue, n=7) and IL-18 KO (orange, n=7) at day 7 after the first K/BxN IgG injection for qRT-PCR analysis. Results represent *Csf2* mRNA expression levels relative to *Gapdh* mRNA levels. Data are shown as the mean ± SEM of values. Statistical analysis performed using a Kruskal–Wallis test did not show a statistical significance.
